# Detection of *Listeria* Species by Conventional Culture-Dependent and Alternative Rapid Detection Methods in Retail Ready-to-Eat Foods in Turkey

**DOI:** 10.4014/jmb.2308.08043

**Published:** 2023-11-16

**Authors:** Emine Dincer

**Affiliations:** Faculty of Health Sciences, Department of Nutrition and Dietetics, Sivas Cumhuriyet University, 58140 Sivas, Turkey

**Keywords:** *Listeria*, listeriosis, ready-to-eat meals

## Abstract

Foodborne pathogens, like *Listeria monocytogenes*, continue to inflict substantial financial losses on the food industry. Various methods for detecting *Listeria* in food have been developed and numerous studies have been conducted to compare the different methods. But, in recent years, new *Listeria* species have been identified, and currently the genus comprises 26 species. Therefore, it would be a more accurate approach to re-evaluate existing detection methods by considering new species. The present investigation involved the analysis of 42 ready-to-eat (RTE) foods, encompassing a variety of food categories, such as mezes, salads, dairy products, and meat products, with the aim of ascertaining the presence of *Listeria*. Among the traditional culture-dependent reference methods, the ISO 11290 method was preferred. The process of strain identification was conducted with the API Identification System. Furthermore, to ascertain the existence of *L. monocytogenes* and *Listeria* spp., the samples underwent additional analysis employing the VIDAS Immunoassay System, ELISA, and RT-PCR methodologies. Thus, four alternative approaches were employed in this study to compare not only the different methods used to determine *Listeria* while taking into account the newly identified *Listeria* species, but also to assess the compliance of retail RTE food items with microbiological criteria pertaining to the genus *Listeria*. Based on the conducted analyses, *L. monocytogenes* was conclusively determined to be present in one sample. The presence of *Listeria* spp. was detected in 30.9% of the samples, specifically in Turkish cig kofte, sliced salami, and salads.

## Introduction

The genus *Listeria* is a highly significant group of microorganisms within the food industry. Currently, it comprises 26 validly published species, plus one species that doesn't yet have a valid status. Six species belonging to this genus have been known for a long time and include *Listeria monocytogenes*, *Listeria innocua*, *Listeria ivanovii*, *Listeria seeligeri*, *Listeria welshimeri*, and *Listeria grayi*. Since its initial discovery in 1926, the genus consisted of only eight species till the year 2010 [1, 2]. Since this date, a multitude of novel species have been discovered, leading to a rapid expansion of the genus. The traditional properties of *Listeria*, such as its ability to survive at 4°C, have lost their universality due to the discovery of new species. With all of this new information about *Listeria*, the genus has been subdivided into two clades: *sensu stricto* (*L. monocytogenes* and similar species to *L. monocytogenes* in terms of characteristics) and *sensu lato* (species more distantly related to *L. monocytogenes*). On the other hand, despite the discovery of new *Listeria* species, whether from the *sensu stricto* or *sensu lato* clade, within the genus, only *L. monocytogenes* is still considered pathogenic for humans [2, 3].

Due to their ability to survive a wide range of environmental conditions, *L. monocytogenes* can be found in different environments, such as food, and environmental and clinical specimens. It proliferates particularly in multifarious food products, including ready-to-eat (RTE) foods, dairy or meat products, vegetables, processed foods and fish [4, 5]. This foodborne pathogen causes disease ranging from mild gastroenteritis to invasive listeriosis, which can also lead to meningitis, miscarriage, septicemia, encephalitis, and endocarditis. Moreover, invasive listeriosis can be lethal for newborns, pregnant women, the elderly, and people with compromised immune systems [4-6]. The incidence of invasive listeriosis is lower than that of diseases caused by other foodborne pathogens, although the EFSA and ECDC [7] both recently reported that cases of listeriosis have increased over the years.

Although several other food categories have also been identified as possible sources of transmission, most cases of listeriosis are known to be caused by the consumption of RTE foods [3, 4, 8, 9]. Because RTE foods are consumed uncooked, the risk of preserving the viability of pathogens such as *L. monocytogenes* is higher. For this type of food, it is crucial to prevent contamination throughout the entire process, from production to consumption. Furthermore, RTE foods with a neutral or moderately low pH and relatively high a_w_ values are considered to support the growth of *L. monocytogenes*. Many countries like the US, New Zealand, Australia, and Turkey currently have a “zero tolerance” approach for all RTE food. However, despite all the precautions and legal regulations, listeriosis outbreaks due to consumption of RTE foods are still observed all over the world [4, 7, 10-12].

Contemporarily, various methods for detecting *Listeria* spp. or *L. monocytogenes* in food have been developed. Culture-dependent traditional methods, recommended by entities like the U.S. Food and Drug Administration (FDA), the International Organization for Standardization (ISO), and the Association of Official Analytical Chemists (AOAC), are still considered the gold standard. The FDA’s Bacteriological Analytical Manual (BAM), ISO 11290, and the AOAC’s official method are routinely used to isolate and identify *Listeria* species. However, since culture-dependent methods are time-consuming, alternative and rapid detection methods based on different approaches have also been developed over time. These methods can be categorized into immunological, spectroscopic, and molecular methods, as well as microfluidic system-, biosensor- and phage-based methods [13-15]. Immunological methods are based on specific antigen-antibody binding. Enzyme-linked immunosorbent assay (ELISA), enzyme-linked fluorescent assay (ELFA), thermal flow immunoassay and immunomagnetic separation are among the most used immunological methods [13]. Simple polymerase chain reaction (PCR), multiplex PCR, real-time PCR (RT-PCR), real-time nucleic acid sequence-based amplification, oligonucleotide-based microarray, and loop-mediated isothermal amplification are among the most used molecular detection methods. Near infrared spectroscopy, matrix-assisted laser desorption ionization time-of-flight mass spectrometry, and raman spectroscopy are among the most used spectroscopic methods. In recent times, in addition to these methods, sensor systems have also been developed to detect *Listeria*, and these include optical, electrochemical, piezolectic, and cell-based sensors [14-16].

Numerous studies have been conducted to compare existing systems with one another. Various methodologies used to identify *Listeria* species exhibit distinct merits and drawbacks relative to one another. However, it is worth noting that a majority of these investigations were conducted prior to the identification of novel *Listeria* species. Consequently, the conducted analyses did not account for newly discovered species.

In Turkey, foods called ‘meze’, which are in the RTE food category and consumed with alcoholic beverages, such as raki, or eaten at the beginning of the meal, are very popular. Foods in this category are sold in supermarkets and public markets. In the current study, 42 RTE foods (mostly meze and salads) were analyzed to determine the presence of *Listeria* spp. and *L. monocytogenes* by using four different methods, including classical method, ELISA, ELFA, and RT-PCR. The aim herein was to evaluate whether retail RTE food products in supermarkets and public markets comply with microbiological criteria in terms of *L. monocytogenes*, and also to compare different methods used to determine *Listeria*, considering both *sensu stricto* or *sensu lato* clades.

## Materials and Methods

### Pilot Study

In the first stage of the study, commercially sold pasteurized milk, which is known not to contain *Listeria* species, was contaminated in a controlled manner, and all analysis methods used in the study were tested. Purchased milk was divided into 3 parts under sterile conditions and *Listeria monocytogenes* 4b ATCC 19115 was added at concentrations of 1 CFU/ml, 10 CFU/ml, and 100 CFU/ml, respectively. Then, all analyses described in detail below were carried out simultaneously with these controlled contaminated samples.

### Sample Collection

A total of 42 RTE foods including various mezes, salads, meat, and dairy products were randomly purchased from supermarkets and public markets in Eskişehir, Turkey, over a three-month period. As soon as they were purchased, samples were aseptically transported to the laboratory in sterile bags under refrigerated conditions, and then immediately transferred to the pre-enrichment medium. Information obtained from the sellers about the RTE foods used in the study is given in Table 1.

### Isolation and Identification of *Listeria* with Conventional Method

In the present study, the EN ISO 11290-1 [17] guidelines were followed for the isolation of *Listeria* strains by culture-dependent methods. Then, selected colonies were identified by using the API *Listeria* Identification System. Half-Fraser broth (225 ml medium in sterile stomacher bags), Fraser broth (10 ml medium in sterile glass test tubes), API *Listeria* Tests (10 300) were purchasedfrom BioMeriux, Turkey. Agar *Listeria* according to Ottaviani and Agosti (ALOA), Palcam Agar, and TSA-YE agar were purchased from Merck, Turkey. The details of the process steps are as follows:

For the pre-enrichment step, a 25 g sample was added to 225 ml of Half-Fraser broth medium and after homogenization in a stomacher (Smasher BioMerieux, France) for 1 min, the mixture was incubated for 25 ± 1 h at 30°C. Then, for the enrichment step, a 0.1 ml sample was collected and transferred into 10 ml Fraser broth medium and incubated for 24 ± 2 h at 37°C. At the end of the incubation period, samples were inoculated onto ALOA and Palcam Agar plates and incubated for 48–72 h at 37°C. Following that, typical *Listeria* colonies were selected, examined by gram-staining, and grown on TSA-YE agar for the API *Listeria* test. After 24–48 h incubation on TSA-YE agar at 37°C, colonies harvested with a sterile loop were transferred to the API suspension medium. The density of suspension medium was adjusted to equal BioMerieux McFarland 1. Then, after distributing approximately 3 ml of distilled water to the bottom of an incubation box and placing the strip inside it, the prepared suspension medium was distributed to each tube. The lid of the incubation box was closed, and the test kit was incubated for 18–24 h at 37°C. At the end of the incubation period, a drop of ZYM B reagent was added to the DIM test and after 3 min, all reactions were read in accordance with the reading chart. For the evaluation, a 4-digit numerical profile was obtained by using the result sheet according to the administrator’s instructions. Species determinations were made by entering the obtained numerical profile into the apiweb database (V2.0) optimized by the company.

### Listeria spp. Detection with ELISA

To determine *Listeria* spp. in food samples by the ELISA method, a Tecra *Listeria* Visual Immunoassay (Tecra Diagnostic) test kit was used. Since this commercially available kit requires pre-enrichment and enrichment steps prior to analysis, the samples prepared for determination by classical method as described before were also used in this study. At the 24th h of incubation in the enrichment step in Fraser broth medium, 1 ml samples were taken and used for ELISA analysis. Analysis was performed using the solutions provided with the kit, according to the administrator's instructions. Briefly, a 50 μl sample additive and a 1 ml sample were added to a test tube and kept in a water bath for 15 min at 100°C. Meanwhile, for each sample, 1 well was placed in the holder. Also 2 wells were added in the holder for positive and negative controls. For each sample, 200 μl of heat-treated sample was transferred into individual wells. Then, 200 μl of positive control solution and 200 μl of negative control solution were added to positive and negative control wells, respectively. All wells were covered with parafilm and incubated for 30 min at 37°C. After that, the wells were inverted to discard the content and then washed 3 times with intensive washing solution. Then, 200 μl of conjugate was added to the cells. All wells were covered with parafilm, and after incubation for 30 min, the content was discarded again and washed 4 times. In the next step, 200 μl of substrate was added to the cells and kept for 15 min at room temperature. At the end of the incubation period, both the color change in the wells was evaluated visually using the color chart (1 and 2 negative, 3, 4 and 5 positive) and the results were recorded and the absorbance at 414 nm was recorded with an ELISA reader.

### *L. monocytogenes* Detection with ELFA

To determine *L. monocytogenes* in food samples by the ELFA method, a VIDAS Immunoassay System (BioMerieux) was used. This system is similar to the determination of *Listeria* spp. with the ELISA method, since it also requires pre-enrichment and enrichment steps prior to analysis and the samples in the enrichment step, prepared for determination by the classical method, were used. At the 24th h of incubation in the enrichment step in Fraser broth medium, 500 μl samples were taken and transferred to a *Listeria monocytogenes* II (LMO2, BioMerieux) test kit. In addition to the samples, standard samples called S1, a positive control called C1, and a negative control called C2 were added to the wells of the strip in the order of identification on the VIDAS device. Then, the strips were inserted into the device and the test was conducted according to the administrator's instructions.

### Listeria spp. Detection with RT-PCR

For determination of *Listeria* spp. in food samples by RT-PCR, in the first step DNA extraction was performed using a Food DNA Extraction Kit (Genoks GFJ-DNA 022, Turkey) and used as a template for RT-PCR analysis. For the extraction, as in the other determination test, the samples in the enrichment step prepared for determination by the classical method were used. At the 24^th^ h of incubation in the enrichment step in Fraser broth medium, a 2 ml sample was taken and centrifuged at 12 rpm for 2 min. The obtained pellet was used for DNA isolation. All process steps were carried out with the ready-made solutions included in the kit according to the instructions of the administrator.

For RT-PCR, a *Listeria* spp. RT-PCR kit (Genoks GFJ-420) was used. Texas Red dye was used for scanning. Analysis was carried out, as recommended by the manufacturer, in 50 μl reaction volume containing 35 μl of RT-PCR master mix, 5 μl of *Listeria* spp.-specific probe, 5 μl template DNA, and 5 μl dH2O. Analysis conditions consisted of initial denaturation for 2 min at 94°C, followed by 40 cycles of denaturation for 45 s at 94°C, annealing for 45 s at 60°C, and extension for 45 s at 72°C.

### Statistical Analysis

The data obtained from the study were analyzed by using the SPSS 23.0 program. Man-Whitney test, one of the non-parametric tests, was used to compare the *Listeria* detection methods, including conventional culture-dependent methods, ELISA, and RT-PCR.

## Results and Discussion

To determine the presence of pathogenic microorganisms in food, numerous alternative detection methods have been developed over time. Among these methods, some immunological techniques such as ELISA and ELFA, and some molecular methods like RT-PCR, are considered widely accepted due to their ease of application, relative cost-effectiveness, and long-standing use. As a result, these techniques have been accepted as the most commonly preferred methods along with the traditional culture-dependent methods [14, 15]. Therefore, in the current study, in addition to the traditional culture-dependent reference method, EN ISO 11290-1, these three methods were chosen based on their widespread acceptance and practicality.

**Fig. 1:**
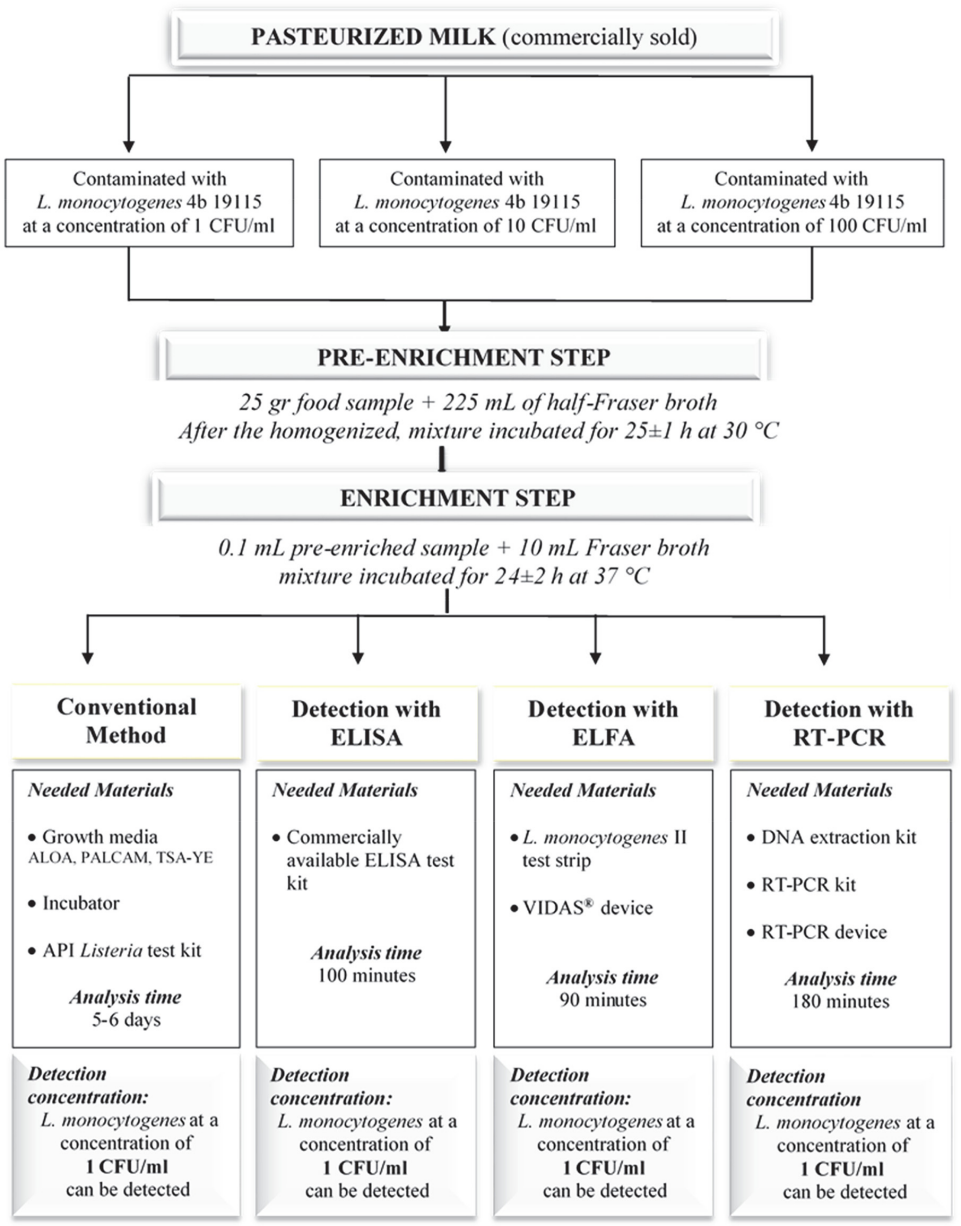
Study design.

In the first stage of the present study, pasteurized milk sample was contaminated in a controlled manner and preliminary trials were carried out for the four different analysis methods, which included the classical method, ELISA, ELFA, and RT-PCR. As a result of the preliminary trials, it was observed that correct results were obtained with all methods used, even when the pasteurized milk sample was contaminated with *L. monocytogenes* at a concentration of 1 CFU/ml. Thus, the methods were confirmed to have been applied correctly. The study design and results of the preliminary trials were presented in Fig. 1. After the preliminary studies, 42 RTE foods sold in supermarkets and public markets in Eskişehir, Turkey was analyzed for the presence of *Listeria* species using more than one method and the results were presented in Table 2.

Today, a wide variety of sensitive, reliable, and validated methods are available for the determination of *Listeria* in foods. Of these methods, FDA BAM, ISO 11290, and the AOAC’s official methods are currently accepted as reference methods worldwide, and all other methods are validated through comparison with these methods [15, 18]. Even so, the reference methods mentioned also have some limitations. Besides being time-consuming and laborious, one of the main problems with these methods are the possibility of false-negative results. It is known that various *sensu stricto* species, such as *L. seeligeri* and *L. ivanovii*, cannot be detected from time to time if the samples contain low levels and/or damaged cells [3, 14]. Moreover, several recent studies have shown that some newly identified *Listeria* *sensu lato* species may not be determined by the ISO method [19]. In the present study, the presence of *Listeria* was detected in 13 food samples by the ISO 11290 method. On the other hand, ELISA and RT-PCR analyses showed the presence of *Listeria* in 18 food samples, and the samples containing *Listeria* spp. showed identical results with the two methods. As a result of the statistical analysis, no significant difference was found between the conventional culture-dependent method, ELISA, and RT-PCR (*p* > 0.05). In the analysis performed by the conventional method, the presence of *Listeria* could not be detected in 5 of these 18 samples mentioned. The other 13 samples agreed with the samples in the ELISA and RT-PCR analyses. As described above, a false-negative result may have been seen for the 5 samples by the ISO 11290 method, due to either insufficient cell presence, cell damage, or the presence of *Listeria* *sensu lato* species. Furthermore, similar results can be obtained in the case of contamination with viable but non-culturable (VBNC) bacteria. On the other hand, the difference in the results obtained from the conventional culture-dependent and alternative methods in the present study may be due to other reasons. Since *Listeria* detection by alternative and rapid detection methods like ELISA and RT-PCR is independent of strain viability, dead cells can also be detected with these systems [13, 20]. Similarly, in the present study, it was possible to find dead cells and get false-positive results in 5 samples by ELISA or RT-PCR.

The Tecra *Listeria* VIA test kit used in this study is a sandwich configuration-based ELISA system and is suitable for detecting *Listeria* spp. in foods. This kit has a very high accuracy rate and has been approved by the AOAC as a colorimetric polyclonal immunoassay screening method. However, various studies have shown that the system can give false-positive and false-negative results, albeit rarely. Noah *et al*. [21], to evaluate commercial ELISA kits, analyzed 178 samples using both commercial Tecra *Listeria* VIA kits and BAM reference methods. At the end of the study, the researchers determined the presence of *Listeria* in 38 and 40 samples, by the BAM and Tecra methods respectively, and although they gave more false-positive results, the researchers recommended the Tecra *Listeria* VIA kit as an alternative to the BAM method as rapid screening procedures. Vanderlinde and Grau [22] investigated the presence of *Listeria* spp. in 170 samples and reported 2 false-positive reactions in their analysis by Tecra *Listeria* VIA test.

The possibility of false-positive and false-negative results is not seen only in immunological methods such as ELISA. Similar situations are also observed in nucleic acid-based methods, such as RT-PCR, whose validity and reliability have been proven. Aznar and Solis [18] screened the presence of *L. monocytogenes* in 225 food samples by three methods and reported 22, 23, and 60 positive samples by ISO 11290-1, VIDAS LMO, and PCR methods, respectively. Netschajew *et al*. [23] evaluated RT-PCR and classical culturing methods for detecting *L. monocytogenes* in 50 vacuum-packed meat products and reported positive results in only a few samples by conventional methods; however, they detected the presence of *L. monocytogenes* in 32 samples by RT-PCR. Barbau-Piednoir *et al*. [24] reported that they developed a SYBR Green qPCR system that works with 98% accuracy for the detection of *Listeria* spp. in foods.

In the reference methods, identification of presumptive *Listeria* colonies is performed by morphological, biochemical, and physiological tests. In these types of analysis, after the isolation to shorten the total analysis time, various commercial rapid identification tests like the API *Listeria* identification system are often preferred [13, 14, 18]. Similarly, in the present study, after purification of *Listeria* strains from ALOA or Palcam agar medium by the conventional method, the identification of strains was performed with the API *Listeria* test. As a result, 6 strains were identified as *L. innocua*, 2 were *L. grayi*, 1 was *L. seeligeri*, 1 was *L. ivanovii*, and 1 strain was *L. monocytogenes*. The strains found in samples 2 and 13 could not be identified. Although the API *Listeria* kit is a highly accurate method, in the test, the results are evaluated according to the color change in the first stage, based on qualitative observation. This situation increases the margin of error during the evaluation of the results and may lead to misidentification or failure to identify strains [25]. Carlin *et al* [2] reported that *Listeria* API analysis misidentified *L. swaminathanii* as *L. monocytogenes*. Aznar and Solis [18] isolated possible *Listeria* strains from 225 food samples with the ISO-11290 method, and identified the strains with the API Lis system. After confirming the identity of the strains with specific PCR, the researchers reported 5 false-positive and 7 false-negative identification results with API.

Since *L. monocytogenes* is the only species in the genus *Listeria* to be considered a human pathogen, most of the developed methods are for the direct detection of *L. monocytogenes* in foods. VIDAS Immunoassay system is suitable for determining both *Listeria* spp. and *L. monocytogenes* through different kits developed [26]. VIDAS LMO2 kit has been developed for the direct detection of *L. monocytogenes* and validated by ANFOR certification [27]. Although this system is now considered an effective alternative to traditional methods [28, 29], the accuracy of the system is recognized as not always 100% and it is recommended that positive results be confirmed with conventional methods [26, 30]. In a study that analyzed 295 foods, 11 false-positive results were obtained in the assay of *L. monocytogenes* with VIDAS LMO2 kits and researchers reported that the specificity of VIDAS LMO2 was 0.96 and the sensitivity 0.73 [30]. A similar result was also observed in the present study. One strain isolated from one sample (sample no. 28) was identified as *L. monocytogenes* by the API Lis kit. However, analysis with VIDAS Immunoassay system showed the presence of *L. monocytogenes* in two samples (samples no. 15 and 28). Colonies in sample no. 15, whose presence of *L. monocytogenes* was determined by VIDAS, were identified as *L. innocua* by API test. For this study, with the available data, it is not possible to say which test results are correct. False-positive results may be obtained with VIDAS analysis, and species can be misidentified by API test. Moreover, there is a third possibility; selective media preferred for analysis and *Listeria* species present in a sample can affect the detection sensitivity. It has been reported in the literature that selective agents such as acriflavine added to Fraser broth may have harmful effects such as stress or cell damage on *L. monocytogenes*. In the pre-enrichment step of the ISO 11290 method, an attempt to solve this problem was made by adding a semi-concentration selective agent into Half-Fraser broth medium. Besides, in Fraser broth medium, *Listeria innocua* has been shown to inhibit the development of *L. monocytogenes* 1/2a [14, 20]. Similarly, in this study, the presence of *L. innocua* in sample no. 15 may have suppressed the isolation of *L. monocytogenes* by culture-dependent methods, but positive results may have been obtained with this system since the VIDAS system detects antigens rather than living cells.

All the results obtained from the four different methods were evaluated together, and in the present study, 30.9%of analyzed RTE foods with certainty contained *Listeria* species, especially *L. innocua*. Additionally, it is noteworthy that *Listeria* species are found especially in Turkish cig kofte from mezes, sliced salami from meat products and generally in salads. The presence of *L. monocytogenes* is certain in only one of the 42 RTE foods. Compared with existing literature data, in the present study, the rate of *L. monocytogenes* is lower. Bilgin *et al*. [31] analyzed 100 samples of salad and appetizers from retailers in Istanbul and reported that they found *L. monocytogenes* in 9% of the samples. Bustamante *et al*. [32] analyzed 400 RTE artisanal food samples requiring minimal or moderate process and reported *L. monocytogenes* in 7.5% of samples. Łepecka *et al*. [12] analyzed 30 different RTE salads sold in Polish markets and reported *L. monocytogenes* in 33.3% of the salads. From the perspective of food hygiene, it should be noted that the presence of *Listeria* species may indicate potential risk of contamination with *L. monocytogenes* [13, 20]. Therefore, even if the *L. monocytogenes* rate was found to be low, it is clear that these products are risky for *Listeria*.

## Conclusion

The conventional culture-dependent techniques employed for the identification of *Listeria* species continue to be regarded as the benchmark method. Nonetheless, the limitations of culture-dependent methods, particularly in detecting *sensu lato* *Listeria* species, and the incapability to detect non-viable/damaged cells, may lead to differences in the results obtained from culture-dependent and other frequently preferred methods. As in this study, the available data obtained from one culture-dependent method and one alternative method like ELISA can be insufficient, and further analyses may be needed to determine which of the different methods used gives the correct result. Due to the risk of misleading results, within species-based identification, it is crucial to use molecular-based techniques to validate the identification of strains following isolation. This type of approach can ensure a definitive and unequivocal confirmation, eliminating any uncertainties. In this context, there is still a need to develop fast and reliable methods for detecting all *Listeria* species and differentiating them at the species level.

## Figures and Tables

**Fig. 2:**
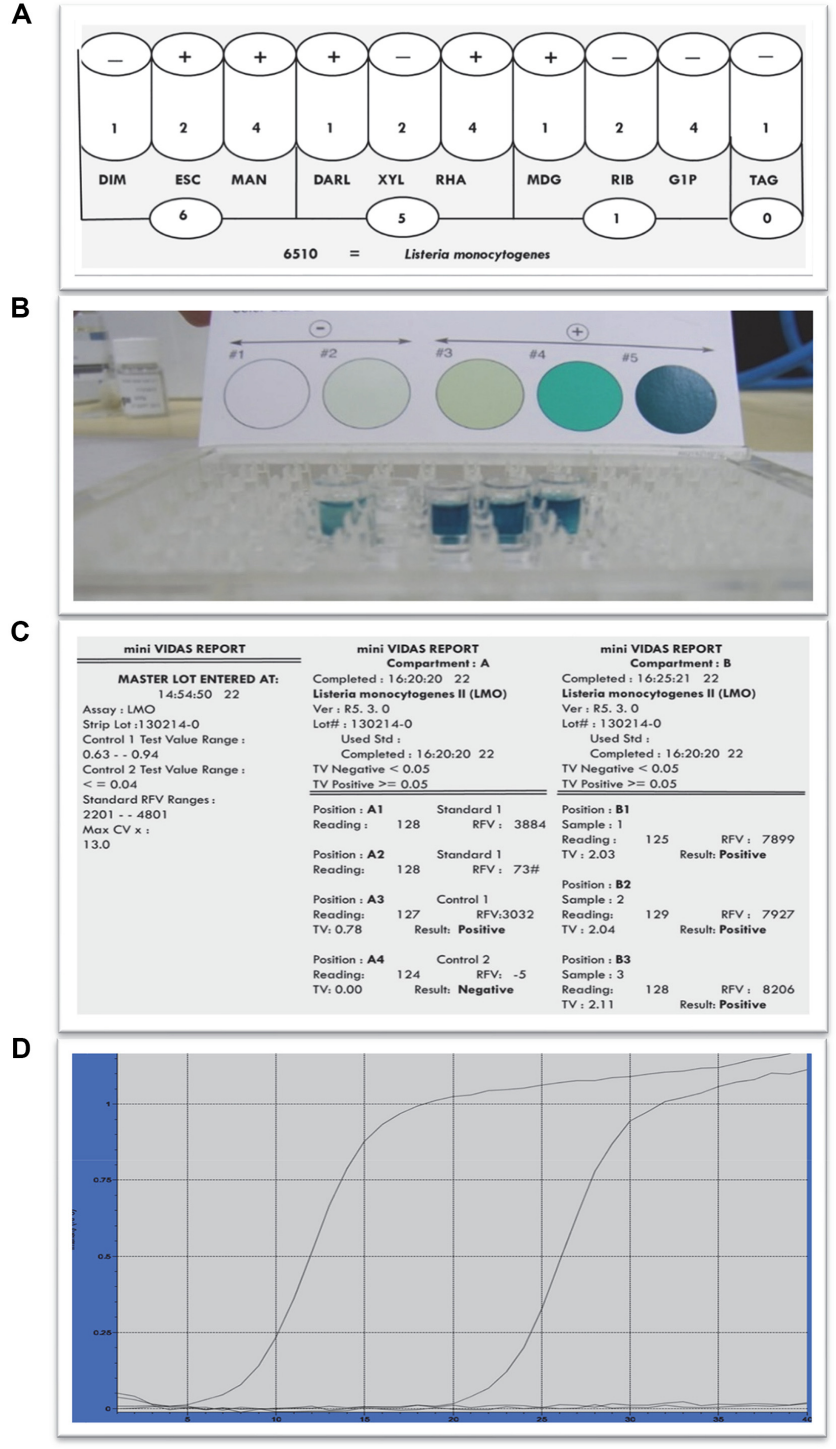
Results of preliminary trials. (**A**) The 4-digit numerical profile and the identified species resulting from the identification of colonies with the API test kit, after isolation with conventional method according to EN ISO 11290-1 standards from a pasteurized milk sample to which *Listeria monocytogenes 4b 19115* was added at a concentration of 1 CFU/ml. (**B**) ELIZA preliminary study results. Wells from left to right;1: positive control, 2: negative control, 3: pasteurized milk contaminated *L. monocytogenes* at a concentration of 1 CFU/ml, 4: pasteurized milk contaminated *L. monocytogenes* at a concentration of 10 CFU/ml, 5: pasteurized milk contaminated *L. monocytogenes* at a concentration of 100 CFU/ml. (**C**) VIDAS preliminary test results. Component A contains standards, positive control and negative control, while component B contains samples to be tested. In component B; B1 pasteurized milk contaminated *L. monocytogenes* at a concentration of 1 CFU/ml, B2: pasteurized milk contaminated *L. monocytogenes* at a concentration of 10 CFU/ml, B3: pasteurized milk contaminated *L. monocytogenes* at a concentration of 100 CFU/ml. (**D**) RT-PCR preliminary test results; The graph showing a logarithmic increase from the 5th cycle is the positive control. The graph showing a logarithmic increase from the 20th cycle is the reaction established with gDNA isolated from contaminated milk, and the graph showing no increase is the reaction established with *E. coli* gDNA.

**Table 1:** Types of food included in the study and some information about them.

Mezes	*Sold in portions according to customer demand and unpackaged*
Turkish cig kofte	Turkish cig kofte (raw meatball) is prepared by kneading ground meat and bulgur with tomato paste, red pepper paste, pomegranate syrup and various spices for about an hour with the addition of hot water.
Authentic Turkish Ezme	Authentic Turkish Ezme is prepared by mixing the peeled and very finely chopped tomatoes (red and sun ripened), onions, green pepper and red pepper with paprika, sumac, salt, chopped parsley and mint.
Tarator	Tarator is a kind of mezze made by mixing bread crumbs, walnuts olive oil and lemon juice, olive oil, garlic and vinegar.
Humus	Hummus is prepared by mixing tahini, chickpeas, garlic, salt, cumin, red pepper, lemon juice and olive oil, until a thick-smooth paste.
Muhammara	Muhammara is a kind of mezze prepared by mixing roasted red pepper (mashed), stale bread (shredded), walnuts (finely pulse), garlic (grated), tomato paste, cumin, salt, olive oil and lemon juice until a smooth paste.
Haydari	Haydari is one of the most popular mezze in Turkey prepared by well mixing yoghurt, fullfat feta cheese (mashed), dill (finely chopped) garlic (grated), mint and salt.
Lentil meatballs	Lentil meatballs is prepared by kneading boiled red lentils and fine bulgur with tomato paste, finely chopped parsley, finely chopped green onion, various spices, salt and onions sautéed in olive oil.
Salads	*Sold in portions according to customer demand and unpackaged*
Russian Salad Italian Salad American Salad	In Turkey, these salads are basically prepared by adding mayonnaise and yogurt to a mixture of potatoes (boiled and chopped), carrots (boiled and chopped), peas (boiled), canned corn and thinly sliced pickles. The difference between 3 salads in the food sector in Turkey is generally as follows. American salad is made with vegetables only. Russian salad includes cold cuts of meat in addition to vegetables. Italian salad, on the other hand, includes julienne-cut salami in addition to vegetables.
Mushroom Salad	In Turkey this salad is mostly prepared by mixing boiled mushrooms, roasted red pepper (chopped) pickled gherkins (chopped), dill, canned corn, lemon juice, pomegranate syrup salt and olive oil.
Roasted Eggplant Salad	In Turkey this salad is prepared by mixing the roasted and peeled eggplant (cut cubes), roasted and peeled bell pepper (finely sliced) tomato (finely chopped), onion (finely chopped), parsley (finely chopped), lemon juice, salt, black pepper, paprika and olive oil.
Meat products	
Salami Ham Chicken doner Sliced Sausage	These products, which can be produced by different brands, are sold packaged and sliced.
Dairy products	
Yoghurt Cream Curd cheese	These products, which can be produced by different manufacturers, are sold in portions/ grams according to customer demand and unpackaged

**Table 2:** Listeria detection using four different methods.

		ISO 11290 and API Listeria test	ELİZA	VIDAS	RT-PCR
1	Turkish cig kofte	*L. grayi* (6120)	+	-	+
2	Authentic Turkish Ezme	ND	+	-	+
3	Tarator	*Listeria seeligeri* (2310)	+	-	+
4	Russian Salad	*Listeria innocua* (7110)	+	-	+
5	Italian Salad	*Listeria ivanovii* (2130)	+	-	+
6	Condensed yoghurt	-	-	-	-
7	Tarator	-	-	-	-
8	Authentic Turkish Ezme	-	-	-	-
9	Russian Salad	-	-	-	-
10	Curd cheese	-	+	-	+
11	Humus	-	-	-	-
12	Curd cheese	-	-	-	-
13	Purslane with yogurt	*Listeria innocua* (7110)	+	-	+
14	Haydari	*Listeria grayi* (7330)	+	-	+
15	American Salad	*Listeria innocua* (7110)	+	+	+
16	Muhammara	*ND*	+	-	+
17	Italian Salad	-	+	-	+
18	Sliced Salami (with pistachio)	-	-	-	-
19	Chicken Ham	-	+	-	+
20	Turkey Ham (with dill)	-	+	-	+
21	Chicken Ham with vegetables	-	-	-	-
22	Hungarian Salami	-	-	-	-
23	Chicken doner	-	-	-	-
24	Russian Salad	-	-	-	-
25	Haydari	-	-	-	-
26	Authentic Turkish Ezme	-	-	-	-
27	Roasted Eggplant Salad	-	-	-	-
28	Sliced Salami	*L. monocytogenes* (6510)	+	+	+
29	Hungarian Salami	-	-	-	-
30	Turkey Salami	*L. innocua* (7510)	+	-	+
31	Sliced Sausage	-	-	-	-
32	Turkish cig kofte	*Listeria innocua* (7510)	+	-	+
33	Chicken doner	-	+	-	+
34	Sliced Salami (with pistachio)	-	-	-	-
35	Russian Salad	*Listeria innocua* (7510)	+	-	+
36	Haydari	-	-	-	-
37	Cream	-	-	-	-
38	Humus	-	-	-	-
39	Tarator	-	-	-	-
40	Mushroom Salad	-	-	-	-
41	Lentil meatballs	-	-	-	-
42	Condensed yoghurt	-	-	-	-
